# *Leishmania* exposure in dogs from two endemic countries from New and Old Worlds (Brazil and Portugal): evaluation of three serological tests using Bayesian Latent Class Models

**DOI:** 10.1186/s13071-022-05328-1

**Published:** 2022-06-13

**Authors:** Carla Maia, Deborah Bittencourt Mothé Fraga, José Cristóvão, Lairton Souza Borja, Manuela da Silva Solcà, Lenea Campino, Patrícia Sampaio Tavares Veras, Luzia Gonçalves

**Affiliations:** 1 Global Health and Tropical Medicine (GHTM), Instituto de Higiene e Medicina Tropical (IHMT), Universidade NOVA de Lisboa (UNL), Lisbon, Portugal; 2 Medical Parasitology Unit, IHMT-UNL, Lisbon, Portugal; 3 Laboratório de Interação Parasito-Hospedeiro e Epidemiologia, Instituto Gonçalo Moniz, FIOCRUZ, Salvador, Bahia, Brazil; 4 Departamento de Medicina Veterinária Preventiva e Produção Animal, Escola de Medicina Veterinária e Zootecnia, Universidade Federal da Bahia, Salvador, Bahia, Brazil; 5 Instituto de Ciência e Tecnologia de Doenças Tropicais, INCT-DT, Bahia, Brazil; 6 International Public Health and Biostatistics Unit, IHMT-UNL, Lisbon, Portugal; 7 Centro de Estatística e Aplicações da, Universidade de Lisboa, Lisbon, Portugal

**Keywords:** Bayesian latent class models, Diagnosis accuracy, Dog, *Leishmania infantum*, Prevalence, Sensitivity, Serology, Specificity

## Abstract

**Background:**

Zoonotic leishmaniosis caused by *Leishmania infantum* is endemic in several countries of the Mediterranean Basin, Latin America, and Asia. Dogs are the main hosts and reservoirs of human infection. Thus, from a One Health perspective, early diagnosis of *Leishmania* infection in dogs is essential to control the dissemination of the parasite among other dogs and to humans. The aim of this study was to estimate the diagnosis accuracy of three serological tests to detect antibodies to *Leishmania* in dogs from two endemic settings using Bayesian latent class models (BLCM).

**Methods:**

A total of 378 dogs from two Portuguese and Brazilian endemic areas of leishmaniosis (194 animals from Portugal and 184 from Brazil) were screened. Detection of anti-*Leishmania* antibodies was performed using two commercial ELISA (*L. infantum* IgG-ELISA^®^ and EIE-LVC^®^) and a rapid immunochromatographic test (DPP-LVC^®^). Bayesian latent class models were used to estimate *Leishmania* infection prevalence, together with sensitivities and specificities of the three diagnostic tests, in the two dog populations simultaneously. Predictive values were also calculated. Credibility intervals (CI) were obtained, considering different types of prior information.

**Results:**

A posterior median *Leishmania* seroprevalence of 13.4% (95% CI 9.0–18.7) and of 21.6% (15.0–28.3) was estimated to the Portuguese and Brazilian dog subpopulations, respectively. The Bayesian analysis indicated that all tests were highly specific (specificity above 90%), and that the DPP-LVC^®^ was more sensitive (96.6%; 83.1–99.9) than both ELISAs in the Portuguese subpopulation, while in the Brazilian subpopulation, EIE-LVC^®^ and *L. infantum* IgG-ELISA^®^, had the highest sensitivity (88.2%; 73.7–97.0) and specificity (98.7%; 95.1–99.9), respectively.

**Conclusions:**

In general, the levels of diagnosis accuracy of the three serological tests to detect *Leishmania* antibodies assessed by BLCM indicate their utility in canine epidemiological studies. The same approach should be used to assess the performance of these techniques in the clinical management of infected and sick dogs using representative samples from the wide spectrum of clinical situations, namely from subclinical infection to manifest disease. The low positive predictive value of the serological tests used in the current protocol of the Brazilian Ministry of Health suggests that they should not be used individually and may not be sufficient to target reservoir-based control interventions.

**Graphical Abstract:**

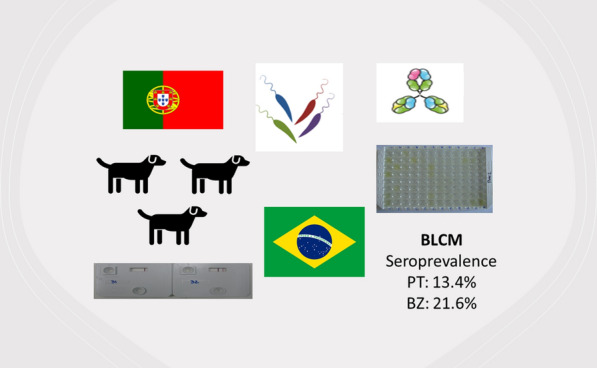

**Supplementary Information:**

The online version contains supplementary material available at 10.1186/s13071-022-05328-1.

## Background

Zoonotic leishmaniosis caused by the protozoan *Leishmania infantum* (syn. *L. chagasi*) is a phlebotomine sand fly-borne disease endemic in several countries of the Mediterranean Basin, Latin America, and Asia. Dogs are the principal hosts and reservoir hosts of human infection [1]. The outcome of *L. infantum* infection ranges from the absence of signs to a severe deadly disease, with the percentage of subclinical infections in endemic areas being much higher than the percentage of clinical disease [2]. Despite the absence of clinical signs, asymptomatic dogs serve as a source of infection for the vectors [3]. Therefore, from a public health and animal health perspective, early confirmation of *Leishmania* infection should be attempted to promote proper management [2, 4].

In clinical and epidemiological studies, enzyme-linked immunosorbent assay (ELISA) and rapid immunochromatographic tests (IRT) are among the most commonly used serological techniques to detect the presence of specific anti-leishmanial antibodies [5]. ELISA technique provides an optical density through an automatic plate reader allowing the quantification of antibody levels, though the sensitivity and specificity are largely dependent of the kind of antigen used [5, 6]. IRTs are easy to perform and to interpret, being ideal for use in the field and in clinical practice; however, IRT only provides a qualitative result which often needs to be confirmed by a quantitative technique; moreover, and although the specificity of these tests is acceptably high, the sensitivity is generally low, particularly in infected subclinical dogs [5, 7].

Brazil is one of the countries reporting > 90% of human visceral leishmaniosis (VL) cases caused by *L. infantum* (https://www.who.int/news-room/fact-sheets/detail/leishmaniasis). Serological screening and euthanasia of seropositive dogs are one of the pillars of the VL control and surveillance program of the Brazilian Ministry of Health [8]. Since 2011, dog culling has been based on the results of a screening test, an IRT (Dual Path Platform canine visceral leishmaniosis test: DPP-LVC^®^), and a confirmatory test, an ELISA (ELISA canine visceral leishmaniosis test; EIE-LVC^®^) [9]. In Portugal, human VL is a hypoendemic disease [10], and reservoir control is limited to mandatory diagnosis and clinical resolution of all dogs considered suspicious for being infected by municipal veterinarians during the vaccination campaign against rabies (Decree-Law no. 19/2020, order 1254). In the first national canine survey carried out in Portugal, an overall seroprevalence of *Leishmania* infection of 6.3% was obtained in dogs attending veterinary clinics, with some regions reaching 17% [11].

As mentioned above, serological methods are among the most common diagnostic techniques used to investigate infection in both subclinical infected and sick dogs [7, 12]. However, there is no serological test with 100% sensitivity and 100% specificity to unambiguously classify an animal as infected. Thus, the use of a reference test will lead to inaccurate estimates with serious epidemiological and clinical consequences: on one hand, false-negative results imply that infected dogs are not detected, contributing to the maintenance of the life cycle of *Leishmania*, delaying clinical management and adequate treatment and worsening the prognosis. On the other hand, false-positive results may lead to the unnecessary killing or treatment of uninfected dogs. In the absence of a gold standard and to avoid the bias associated with an imperfect test, the diagnostic performance of two or more tests can be estimated without knowing the true infection status of the study individuals by latent class analysis (LCA), particularly by a Bayesian approach which may include informative or non-informative prior distributions to represent previous knowledge on parameters to be estimated [13, 14]. However, and so far, LCA has been used in few studies to assess the performance of *Leishmania* diagnostic tests in dogs [15–19] as well as in other vertebrate hosts [20–22].

This study aimed to estimate the diagnostic accuracy of three serological tests to detect antibodies against *Leishmania* in canine sera obtained in two *L. infantum* endemic regions, one in Portugal and the other in Brazil, using Bayesian latent class models (BLCM), without admitting a reference test, estimating simultaneously the prevalence values in each setting.

## Methods

### Canine serum samples

Serum samples from a total of 378 dogs (194 from Portugal and 184 from Brazil) obtained in previous epidemiological studies [16, 23] were analyzed. Portuguese dogs were from four private kennels of the Metropolitan Lisbon region, and domiciled Brazilian dogs were from the municipality of Camaçari, located in the State of Bahia. Samples were randomly selected from previous studies without regard to the clinical status of the animals (i.e. presence or absence of clinical signs compatible with leishmaniosis), and the sample size was based on the availability and the feasibility of the three serological tests under economic restrictions. Initially, 200 dogs from each setting were fixed to this study, using Microsoft Excel^®^ program to randomly select ID codes from a database of previous studies in our laboratories. Due to some laboratorial constraints, the final samples were reduced to 194 from Portugal and 184 from Brazil. All animals were from endemic areas for leishmaniosis caused solely by *L. infantum.* Peripheral blood was obtained by venipuncture from each animal, and serum samples were stored at −20 °C until use in serological analyses.

### Detection of anti-Leishmania antibodies

Detection of anti-*Leishmania* antibodies was performed by two commercial ELISAs, *Leishmania infantum* IgG-ELISA^®^ (Bordier Affinity Products SA, Switzerland) and EIE-LVC^®^ (Bio-Manguinhos, Brazil), and by a rapid immunochromatographic test DPP-LVC^®^ (Bio-Manguinhos) following manufacturer’s recommendations. ELISA result was considered positive when the absorbance of the analysed sample was higher than the absorbance of the weak positive control serum provided with the kit. The *Leishmania infantum* IgG-ELISA^®^ and EIE-LVC^®^ cut-offs were 0.260 and 0.215, respectively, according to manufacturer’s instructions. For the DPP-LVC^®^, the appearance of two pink lines indicated a positive result while the appearance of only one line in the control indicated a negative result.

### Statistical analysis

BLCM was adopted to evaluate the accuracy of the three diagnostic tests, given the absence of a gold standard. Our model assumed that all pairs of tests are conditionally independent, an assumption that is only expected to be valid if two tests are designed from different biological mechanisms [24].

Several models were built to jointly estimate the parameters (sensitivity and specificity of each diagnostic test, prevalence in each setting). The predictive positive and negative values (PPV and NPV) were also indirectly estimated using the expressions based on sensitivity, specificity, and prevalence values [14, 25]. Bayesian approach may combine information from the collected data and prior information about the parameters. Prior information on the prevalence of infection within each subpopulation was considered from a Uniform distribution in [0, 0.30], based on previous studies [16, 23]. Initially, uniform distributions in [0, 1] were used to express non-informative information for the test accuracy (sensitivity and specificity of the tests across populations). Additionally, uniform distributions in [0.60, 1] for sensitivities and specificities in both settings and beta prior distributions for the sensitivity and specificity of the EIE-LVC^®^ and DPP-LVC^®^, based on some published works, were also considered in several fitted models. Beta distributions were explored, using the EpiTools Program [26].

All analyses were implemented in OpenBUGS, using the R2OpenBUGS and MCMCplots packages [27] in R Program (R Foundation for Statistical Computing, Vienna, Austria). In general, inferences were based on 40,000 iterations, after discarding an initial burn-in of 5000 iterations, then of 5, with convergence assessed by running multiple chains from various starting values [28]. All parameters were estimated, using the median of the posterior distributions, and 2.5% and 97.5% percentiles were used to present the 95% credibility intervals (95% CI), the Bayesian version of the confidence intervals. The deviance information criterion (DIC) was used as measure of the model fitting. Usually, the model with the smallest DIC is the selected to summarize the main findings. However, if competing models differ in DIC by less than three units, the models are not considered statistically different [29, 30]. Convergence diagnostics and autocorrelation were examined by visual plots and measures [31, 32].

STARD-BLCM guidelines were followed to report the results of this Bayesian analysis [33–35] (Additional file 1: Table S1; Additional file 4: Figure S1).

Catterplots were used to show some results in ggplot2 from R Package. Cohen’s kappa coefficients for each pair of the three tests were also obtained, considering their importance in veterinary medicine [17]. This parameter was determined as follows: no agreement (*k* < 0), slight agreement (0 < *k* < 0.2), fair agreement (0.2 < *k* < 0.4), moderate agreement (0.4 < *k* < 0.6), substantial agreement (0.6 < *k* < 0.8) and very good agreement (*k* > 0.8).

## Results

The combination of results obtained by the three serological tests in each population of dogs is showed in Table 1. In the Portuguese and Brazilian populations, 39 (20.1%) and 76 (41.3%) of the dogs were reactive to at least one serological test, respectively. Antibodies to the parasite were detected by the three techniques in 20 (10.3%) dogs from Portugal, while of the 14 animals positive for only one test, 12 were tested using the DPP-LVC^®^. In Brazil, the number of dogs considered seropositive by the three techniques was 25 (13.6%), while the number of those positive by a single technique varied between 24, tested by the *Leishmania infantum* IgG ELISA^®^, and 1, tested by the DPP-LVC^®^.

**Table 1:** Combined results of the three serological tests performed to assess the presence of antibodies against *Leishmania* parasites in two samples—Portuguese and Brazilian dogs

*Leishmania infantum* IgG ELISA^®^	EIE-LVC^®^	DPP-LVC^®^	Portuguese dogs (*n* = 194)	Brazilian dogs (*n* = 184)	Total (*n* = 378)
+	+	+	20	25	45
+	+	−	2	8	10
+	−	+	0	4	4
+	−	−	1	24	25
−	+	+	3	3	6
−	+	−	1	11	12
−	−	+	12	1	13
−	−	−	155	108	263

(+) indicates a positive and (−) indicates a negative test result

*IgG* Immunoglobulin G, *ELISA* enzyme-linked immunosorbent assay, *EIE*-*LVC*^®^ ELISA canine visceral leishmaniosis test, *DPP*-*LVC*^®^ Dual Path Platform canine visceral leishmaniosis test

According to prior information based on uniform distributions [0, 1] for all sensitivities and specificities and uniform distributions [0, 0.30] for prevalence values, after combining with the data information, a posterior median *Leishmania* seroprevalence of 13.4% 95% CI (9.0–18.7) and of 21.6% (15.0–28.3) was estimated to the Portuguese and Brazilian dog populations, respectively (Table 2). In the population of Portuguese dogs, the estimate sensitivity of the three tests was > 85%, with DPP-LVC^®^ showing the highest median value (96.6%) and the smallest uncertainty (95% CI 83.1–99.9). The specificity of all tests was > 92.5%, with both DPP-LVC^®^ and EIE-LVC^®^ showing the highest median value (99.1%) and credibility intervals of (95% CI 96.8–99.9 for EIE-LVC^®^ and 96.6–100.0 for DPP-LVC^®^). The PPV (94.3%) and NPV (99.5%) for DPP-LVC^®^ were also the highest.

**Table 2:** Diagnostic accuracy of the three serological tests, using Bayesian latent class models with non- informative prior distributions, except to prevalence values, for Portuguese and Brazilian dogs, given by posterior median and 95% credibility intervals

	Prevalence median (95% CI)	Serological tests	Sensitivity median (95% CI)	Specificity median (95% CI)	PPV median (95% CI)	NPV median (95% CI)
Portuguese dog population		*Leishmania infantum* IgG ELISA^®^	88.5 (72.5–97.3)	92.5 (88.0–95.9)	64.4 (47.1–79.1)	98.2 (95.1–99.6)
	13.4 (9.0–18.7)	EIE-LVC^®^	85.2 (67.7–95.7)	99.1 (96.8–99.9)	93.3 (78.4–99.4)	97.8 (94.6–99.4)
		DPP-LVC^®^	96.6 (83.1–99.9)	99.1 (96.6–100.0)	94.3 (79.5–99.7)	99.5 (97.2–100.0)
Brazilian dog population		*Leishmania infantum* IgG ELISA^®^	79.9 (61.3–95.9)	98.7 (95.1–99.9)	94.5 (79.3–99.7)	94.7 (88.3–99.1)
	21.6 (15.0–28.3)	EIE-LVC^®^	88.2 (73.7–97.0)	81.8 (74.3–88.1)	56.9 (41.5–71.1)	96.3 (90.6–99.1)
		DPP-LVC^®^	85.7 (70.7–96.0)	90.7 (84.3–95.7)	71.6 (53.7–86.7)	95.9 (90.6–99.0)

Deviance information criterion: 69.96; pD = 10.07

Prior information for prevalence: uniform (0, 0.30)

*CI* credibility intervals, *IgG* immunoglobulin G, *ELISA* enzyme-linked immunosorbent assay, *EIE*-*LVC*^®^ *ELISA* canine visceral leishmaniosis test, *DPP*-*LVC*^®^ Dual Path Platform canine visceral leishmaniosis test, *PPV* positive predictive value, *NPV* negative predictive value

In dogs from the Brazilian population, the sensitivity of the *Leishmania infantum* IgG ELISA^®^ was almost 80% (CI 61.3–95.9). The remain tests presented better values, namely 88.2% (CI 73.7–95.9) obtained with EIE-LVC^®^. Conversely, the lowest specificity (81.8%) and CI 74.3–88.1 were observed with this test. The highest PPV (94.5%) and NPV (96.3%) were obtained for *L. infantum* IgG-ELISA^®^ and EIE-LVC^®^, respectively.

**Fig. 1:**
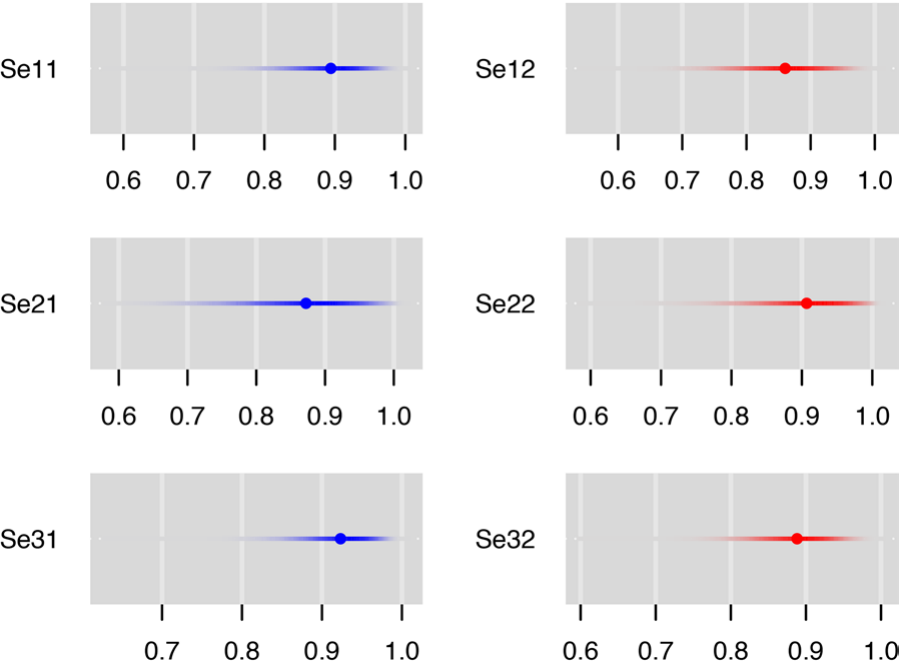
Catterplots for sensitivities of the three diagnostics tests in Portugal (blue) and Brazil (red), using BLCM with informative priors. Se11, Se21, and Se31 are the sensitivities of the *Leishmania infantum* IgG ELISA^®^, EIE-LVC^®^, and DPP-LVC^®^, respectively, in Portugal. Se12, Se22, Se23 are the sensitivities of the *Leishmania infantum* IgG ELISA^®^, EIE-LVC^®^, and DPP-LVC^®^, respectively, in Brazil

No major differences in the posterior estimates were observed when prior information for sensitivities and specificities was used (Additional file 2: Table S2). In general, the selected model would be the one with the smaller DIC, but here DIC values were also very similar. Figure 1 shows this similarity for sensitivities, highlighting the differences between the two settings.

In terms of the agreement levels between diagnostic tests (Additional file 3: Table S3), in Portuguese setting the values were higher compared to Brazil, ranging from a substantial value (*k* = 0.638; *P* < 0.001, for *Leishmania infantum* IgG ELISA^®^ vs. DPP-LVC^®^) to very good agreement (*k* = 0.883, *P* < 0.001, for *Leishmania infantum* IgG ELISA^®^ vs. EIE-LVC^®^).

## Discussion

In endemic areas of zoonotic leishmaniosis caused by *L. infantum*, infected dogs represent a source of parasites to the vectors, thus early detection of *Leishmania* infection in dogs with and without clinical signs is essential to control the dissemination of the parasite to other dogs and humans [2–4]. Due to the absence of a gold standard diagnostic test to assess *L. infantum* infection in dogs presenting or not clinical signs, in the present study a Bayesian latent class analysis was, therefore, performed to estimate the diagnostic performance of three tests for the detection of antileishmanial antibodies in sera from dogs from two endemic areas, one in Portugal and another in Brazil.

The estimated posterior median of *Leishmania* seroprevalence of 13.4% obtained with both models in the dogs from Portugal was similar to the percentage obtained with the total number of canine sera tested with *L. infantum* IgG-ELISA^®^, i.e. 12.5% and 13.9% at the beginning and end of the sand fly season, respectively [23]. The three techniques used in this study were found to be specific for the detection of antibodies against *Leishmania* in the Portuguese population of dogs, with specificity values between 92.5 and 99.1%. Concerning sensitivity, the DPP-LVC^®^ was found to be the best assay for the serodiagnosis of *Leishmania* infection, which together with the highest PPV highlights its relevance as a screening tool to detect exposure to/contact with the parasite. Furthermore, of the 14 animals that were positive for just one test, 12 were tested with this IRT suggesting its diagnostic value in a clinical setting. As the samples used in this study came from an epidemiological survey where dogs were randomly tested, not considering any specific clinical picture, it would be important to assess the diagnostic performance of DPP-LVC^®^ in serum samples from dogs at various stages of the disease. Unfortunately, this IRT, based on the recombinant chimeric protein rK28, resulted from the fusion of *L. infantum* k9, single repeat units of k39 and k26 genes, is available exclusively to Brazilian local public health authorities and official laboratories. Thus, even though it is a practical and fast alternative to the on-site diagnosis of infection, it is unavailable to private veterinarians and diagnostic laboratories. However, the high diagnostic performance of rK28 reported here corroborates the high levels of sensitivity (96%) and specificity (99%) of a rK28-based ELISA developed and evaluated by [36] using classical validation approaches. The higher diagnostic performance of an rK28 antigen-based plasmonic ELISA compared to DPP-LVC^®^ has also been reported recently [37]. The usefulness of this ELISA, whose results can be read with the naked eye, in the serodiagnosis of canine *Leishmania* infection, whether in epidemiological studies or clinical practice, deserves further validation.

The estimate of *Leishmania* seroprevalence for the Brazilian canine population applying non-informative and informative previous distributions for the sensitivity and specificity of the diagnostic tests was 21.6%. This value was, compared to the total of serum samples from which the 184 tested in this study were obtained, higher than the frequency of positive results with DPP-LVC^®^ (16.9%) and lower than that obtained with EIE-LVC^®^ (24 0.9%) [16]. In the present study, the BLCM estimated a sensitivity of 85.7% (70.7–96.0) and a specificity of 90.7% (84.3–95.7) for the DPP-LVC^®^. These findings contrast with those reported in dogs from endemic and non-endemic Argentinian areas (LCA estimated sensitivity of 100% and a specificity of 95.6%, [18]) and with those tested on a pool of sera from Brazilian dogs with and without clinical signs of disease (sensitivity ranging from 90.6 to 97.9%, and specificity ranging from 93.6 to 100%) using classical validation approaches [38–40], reinforcing the bias added using imperfect reference standards. On the other hand, the estimates of BLCM sensitivity 88.2% (73.7–97.0) and specificity 81.8% (74.3–88.1) of the EIE-LVC^®^ obtained in this study were within the results reported by others (sensitivity ranging from 84.2 at 98.96% and specificity ranging from 52.25 to 95.6%) using parasitological diagnoses as reference tests [38, 39, 41]. The use of imperfect tests as a reference can seriously under or overestimate the performance of the diagnostic tests, hampering the diagnosis of *Leishmania* infection in dogs, with consequences for both animal and public health, and this bias is certainly one of the reasons why the culling of seropositive dogs in Brazil is not an effective strategy to reduce the incidence of VL [42]. The VL control and surveillance program currently uses the DPP-LVC^®^ for the official screening of dogs and the EIE-LVC^®^ for confirmation of positive results, with canine euthanasia only being performed when the serological results are positive in both tests [9]. This approach aims to prevent uninfected dogs from being unnecessarily euthanized and infected dogs from remaining undetected in endemic areas. The diagnostic accuracy of this protocol is considered higher than the previous one, as it reduces false-positive results [16]. Although the diagnostic performance of the combination of the two tests has not been evaluated, the higher specificity and PPV of the DPP-LVC^®^ suggest that it could be used as a confirmatory test, as argued earlier [38, 43]. Moreover, and considering the higher PPV of *Leishmania infantum* IgG ELISA^®^ (92.7% versus 65.0% of DPP-LVC^®^ and 50.3% of EIE-LVC^®^), this would be the best choice among the three serological tests evaluated for diagnosing truly positive dogs. Interestingly, of the 36 animals that were positive for just one test, 24 (13.0%) were tested with this ELISA demonstrating its value in terms of detection of canine antibodies against *Leishmania*.

An alternative to the *Leishmania infantum* IgG ELISA^®^ that is not available in the Brazilian market is the use of recombinant antigens in ELISA [5], such as the rK28-based ELISA mentioned above [36, 37]. This alternative would ease the screening of large numbers of samples at a lower cost than IRTs and would minimize the undesirable culling of false-positive dogs. Although both ELISAs were performed with crude antigens, the higher specificity value of the *L. infantum* IgG-ELISA^®^, compared to the EIE-LVC^®^ and the fair agreement between both techniques are likely due to the lower propensity of antigens based on the species responsible for causing infection in dogs to cross-react with other trypanosomatids [38, 44].

Overall, the posterior distributions for each dog population express a slightly better sensitivity of the *L. infantum* IgG-ELISA^®^ and DPP-LVC^®^ in the Portuguese population and a better specificity of the former in the dogs from Brazil. A justification for the overall lower sensitivity of both ELISAs in detecting the presence of parasitic antibodies could be that the cut-off determination is normally done using sick animals and does not consider that antibody levels are distinct and may fluctuate during infection. Our results suggest that a standard cut-off based on the antibody levels of diseased dogs is not the most suitable for all moments of infection, especially when applied to samples collected in epidemiological studies [45]. These differences in test accuracy can also be explained by the possible heterogeneity of *L. infantum* strains or by the presence in the endemic areas of pathogens that can cause cross-reactivity [46]. The difference in the percentage of positive dogs to at least one test between the Portuguese and Brazilian populations may be related to these epidemiological and biological differences, which may also be the reason why the agreement between the three techniques applied to the Portuguese canine samples was substantial or very good but fair or moderate when applied to canine sera from the Brazilian population. Fifty-one of the 70 unmatched serological results occurred in the canine samples from Brazil, evidencing the importance, from a public and animal health point of view, of choosing the serological technique, especially when using a single test. Given that in regions where *Leishmania* species occur sympatrically, a standard cut-off would certainly present a less than desirable performance, the adaptation of the ELISA cut-off could represent a way to overcome misdiagnosis related to cross-reactivity. Furthermore, the regional adaptation of the cut-off together with the use of antigens from the *Leishmania* species and strains known to circulate in each region would certainly improve the accuracy of these serological techniques [46].

## Conclusions

In general, BLCM proved to be a useful tool to assess the performance of diagnostic methods for detecting anti-*Leishmania* antibodies in dogs from two endemic areas for leishmaniosis in the absence of a gold standard. The overall levels of diagnostic accuracy of the three serological tests indicate their usefulness in canine epidemiological studies to be carried out in Portugal or Brazil. However, the low PPV of the two serological tests implemented by current Brazilian Ministry of Health guidelines indicates that they may not be sufficient to target reservoir-based control interventions. The diagnostic accuracy of these techniques for the clinical management of infected and sick dogs should be estimated using Bayesian latent class models.

## Supplementary Information

**Additional file 1: Table S1.** Checklist of standards for reporting the diagnostic accuracy of the present study using Bayesian latent class models.

**Additional file 2: Table S2.** Diagnostic accuracy of the three serological tests, using by Bayesian latent class models with prior informative distributions for Portuguese and Brazilian dogs, given by posterior median and 95% credibility intervals.

**Additional file 3: Table S3.** Cohen's kappa coefficients for each pair of the three tests in the two samples—Portuguese and Brazilian dogs.

**Additional file 4: Figure S1.** Flowchart of the participants according to standards for reporting the diagnostic accuracy of the serodiagnostic techniques tested in the present study using Bayesian latent class models.

## Data Availability

The data supporting the conclusions of this article are included within the article.

## Abbreviations

BLCM: Bayesian latent class models
CI: Credibility intervals
DPP-LVC: Dual Path Platform canine visceral leishmaniosis test
EIE-LVC: ELISA canine visceral leishmaniosis test
ELISA: Enzyme-linked immunosorbent assay
IgG: Immunoglobulin G
IRT: Immunochromatographic rapid test
L: *Leishmania*
LCA: Latent class analysis
VL: Visceral leishmaniosis
