# Supplementary material for: Leishmania exposure in dogs from two endemic countries from New and Old Worlds (Brazil and Portugal): evaluation of three serological tests using Bayesian Latent Class Models
Source: Parasit Vectors. 2022 Jun 13;15:202. doi: 10.1186/s13071-022-05328-1 (PMC9195323; doi:10.1186/s13071-022-05328-1)
Supplement: Supplementary file 1 — Additional file 1: Table S1. Checklist of standards for reporting the diagnostic accuracy of the present study using Bayesian latent class models. [file 13071_2022_5328_MOESM1_ESM.docx]

|  | **Section & Topic** | **No** | **Item** | **Reported on page #** |
| --- | --- | --- | --- | --- |
|  |  |  |  |  |
|  | **TITLE OR ABSTRACT** |  |  |  |
|  |  | **1** | Identification as a study of diagnostic accuracy, using at least one measure of accuracy (such as sensitivity, specificity, predictive values, or AUC) **and Bayesian latent class models** | 1 |
|  | **ABSTRACT** |  |  |  |
|  |  | **2** | Structured summary of study design, methods, results, and conclusions  (for specific guidance, see STARD for Abstracts) | 2 |
|  | **INTRODUCTION** |  |  |  |
|  |  | **3** | Scientific and clinical background, including the intended use and clinical role of the **tests under evaluation** | 3 |
|  |  | **4** | Study objectives and hypotheses, **such as estimation of diagnostic accuracy of the tests for a defined purpose through BLCM** | 4 |
|  | **METHODS** |  |  |  |
|  | *Study design* | **5** | Whether data collection was planned before the **tests** were performed (prospective study) or after (retrospective study) | 4 |
|  | *Participants* | **6** | Eligibility criteria **and description of the source population** | 4-5 |
|  |  | **7** | On what basis potentially eligible participants were identified  (such as symptoms, results from previous tests, inclusion in registry) | 5 |
|  |  | **8** | Where and when potentially eligible participants were identified (setting, location and dates) | 4-5 |
|  |  | **9** | Whether participants formed a consecutive, random or convenience series | Not Applicable |
|  | *Test methods* | **10** | **Description of the tests under evaluation**, in sufficient detail to allow replication, **and/or cite references** | 5 |
|  |  | **11** | Rationale for choosing the **tests under evaluation in relation to their purpose** |  |
|  |  | **12** | Definition of and rationale for test positivity cut-offs or result categories of **the tests under evaluation**, distinguishing pre-specified from exploratory | 5 |
|  |  | **13** | Whether clinical information was available to the performers or readers of **the tests under evaluation** | Not Applicable |
|  | *Analysis* | **14a** | **BLCM model** for estimating measures of diagnostic accuracy | 5-6 |
|  |  | **14b** | **Definition and rationale of prior information and sensitivity analysis** | 5 |
|  |  | **15** | How indeterminate results **of the tests under evaluation** were handled | Not Applicable |
|  |  | **16** | How missing data **of the tests under evaluation** were handled | 5 |
|  |  | **17** | Any analyses of variability in diagnostic accuracy, distinguishing pre-specified from exploratory | Not Applicable |
|  |  | **18** | Intended sample size and how it was determined | 5 |
|  | **RESULTS** |  |  |  |
|  | *Participants* | **19** | Flow of participants, using a diagram | Not Applicable |
|  |  | **20** | Baseline demographic and clinical characteristics of participants | 4-5 |
|  |  | **21** | **Not applicable: the distribution of the targeted conditions is unknown, hence the use of BLCM** |  |
|  |  | **22** | Time interval and any clinical interventions between **the tests under evaluation** | Not Applicable |
|  | *Test results* | **23** | Cross tabulation of the **tests’ results (or for continuous tests results their distribution by infection stage)** | 6 (Table 1) |
|  |  | **24** | Estimates of diagnostic accuracy **under alternative prior specification** and their precision (such as 95% **credible/probability intervals**) | 7 (Table 2 and Table S2) |
|  |  | **25** | Any adverse events from performing **the tests under evaluation** | Not Applicable |
|  | **DISCUSSION** |  |  |  |
|  |  | **26** | Study limitations, including sources of potential bias, statistical uncertainty, and generalisability | 8-11 |
|  |  | **27** | Implications for practice, including the intended use and clinical role of **the tests under evaluation in relevant settings (clinical, research, surveillance etc.)** | 8-11 |
|  | **OTHER INFORMATION** |  |  |  |
|  |  | **28** | Registration number and name of registry | Not Applicable |
|  |  | **29** | Where the full study protocol can be accessed | 11 |
|  |  | **30** | Sources of funding and other support; role of funders | 12 |
|  |  |  |  |  |
