# Supplementary material for: Leishmania exposure in dogs from two endemic countries from New and Old Worlds (Brazil and Portugal): evaluation of three serological tests using Bayesian Latent Class Models
Source: Parasit Vectors. 2022 Jun 13;15:202. doi: 10.1186/s13071-022-05328-1 (PMC9195323; doi:10.1186/s13071-022-05328-1)
Supplement: Supplementary file 2 — Additional file 2: Table S2. Diagnostic accuracy of the three serological tests, using by Bayesian latent class models with prior informative distributions for Portuguese and Brazilian dogs, given by posterior median and 95% credibility intervals. [file 13071_2022_5328_MOESM2_ESM.docx]

**Table S2**. Diagnostic accuracy of the three serological tests, using by Bayesian Latent Class Models with prior informative distributions for Portuguese and Brazilian dogs, given by posterior median and 95% credibility intervals

|  | **Prevalence Median (95% CI)** | **Serological tests** | **Sensitivity**  **Median (95% CI)** | **Specificity**  **Median (95% CI)** | **PPV**  **Median (95% CI)** | **NPV**  **Median (95% CI)** |
| --- | --- | --- | --- | --- | --- | --- |
| Portuguese  dog population |  | *Leishmania infantum* IgG ELISA® | 88.6 (72.7-97.3) | 92.6 (87.9-96.0) | 64.4 (47.7-79.2) | 98.2 (95.2-99.6) |
|  | 13.4 (9.0-18.4) | EIE-LVC® | 85.1 (68.2-95.8) | 99.1 (96.8-99.9) | 93.3 (78.5-99.4) | 97.8 (94.7-99.4) |
|  |  | DPP-LVC® | 96.6 (83.4-99.9) | 99.1 (96.6-100.0) | 94.2 (79.4-99.7) | 99.5 (97.3-100.0) |
|  |  |  |  |  |  |  |
| Brazilian dog population |  | *Leishmania infantum* IgG ELISA® | 79.9 (63.2-95.8) | 98.7 (95.1-99.9) | 94.5 (79.6-99.7) | 94.7 (88.4-99.1) |
|  | 21.5 (15.0-29.2) | EIE-LVC® | 88.2 (73.7-96.9) | 81.9 (74.3-88.2) | 57.0 (41.6-716) | 96.2 (90.5-99.1) |
|  |  | DPP-LVC® | 85.8 (70.7-96.1) | 85.8 (70.7-96.1) | 71.7 (53.9-86.8) | 95.9 (90.4-99.0) |

Deviance Information Criterion: 69.88; pD=10.07

Prior distributions – Prevalence: Uniform (0,0.30); Sensitivities and specificities: Uniform (0.60, 1)

Abbreviations: CI, credibility intervals; IgG, Immunoglobulin G; ELISA, enzyme-linked immunosorbent assay; EIE-LVC®, ELISA canine visceral leishmaniosis test; DPP-LVC® Dual Path Platform canine visceral leishmaniosis test; PPV, positive predictive value; NPV, negative predictive value.
