# Supplementary material for: Leishmania exposure in dogs from two endemic countries from New and Old Worlds (Brazil and Portugal): evaluation of three serological tests using Bayesian Latent Class Models
Source: Parasit Vectors. 2022 Jun 13;15:202. doi: 10.1186/s13071-022-05328-1 (PMC9195323; doi:10.1186/s13071-022-05328-1)
Supplement: Supplementary file 3 — Additional file 3: Table S3. Cohen's kappa coefficients for each pair of the three tests in the two samples—Portuguese and Brazilian dogs. [file 13071_2022_5328_MOESM3_ESM.docx]

**Table S3**. Cohen's kappa coefficients for each pair of the three tests in the two samples - Portuguese and Brazilian dogs

| **Pair of tests** | **Portuguese dogs* (*n*=194)** | **Brazilian**  **dogs***  **(*n*=184)** |
| --- | --- | --- |
| *Leishmania infantum* IgG ELISA® *vs* EIE-LVC® | 0.883 | 0.453 |
| *Leishmania infantum* IgG ELISA® *vs* DPP-LVC® | 0.638 | 0.501 |
| EIE-LVC® *vs* DPP-LVC® | 0.709 | 0.620 |

*All cases: p<0.001

Abbreviations: IgG, Immunoglobulin G; ELISA, enzyme-linked immunosorbent assay; EIE-LVC®, ELISA canine visceral leishmaniosis test; DPP-LVC® Dual Path Platform canine visceral leishmaniosis test.
