# Supplementary figures and images for: Leishmania exposure in dogs from two endemic countries from New and Old Worlds (Brazil and Portugal): evaluation of three serological tests using Bayesian Latent Class Models
Source: Parasit Vectors. 2022 Jun 13;15:202. doi: 10.1186/s13071-022-05328-1 (PMC9195323; doi:10.1186/s13071-022-05328-1)

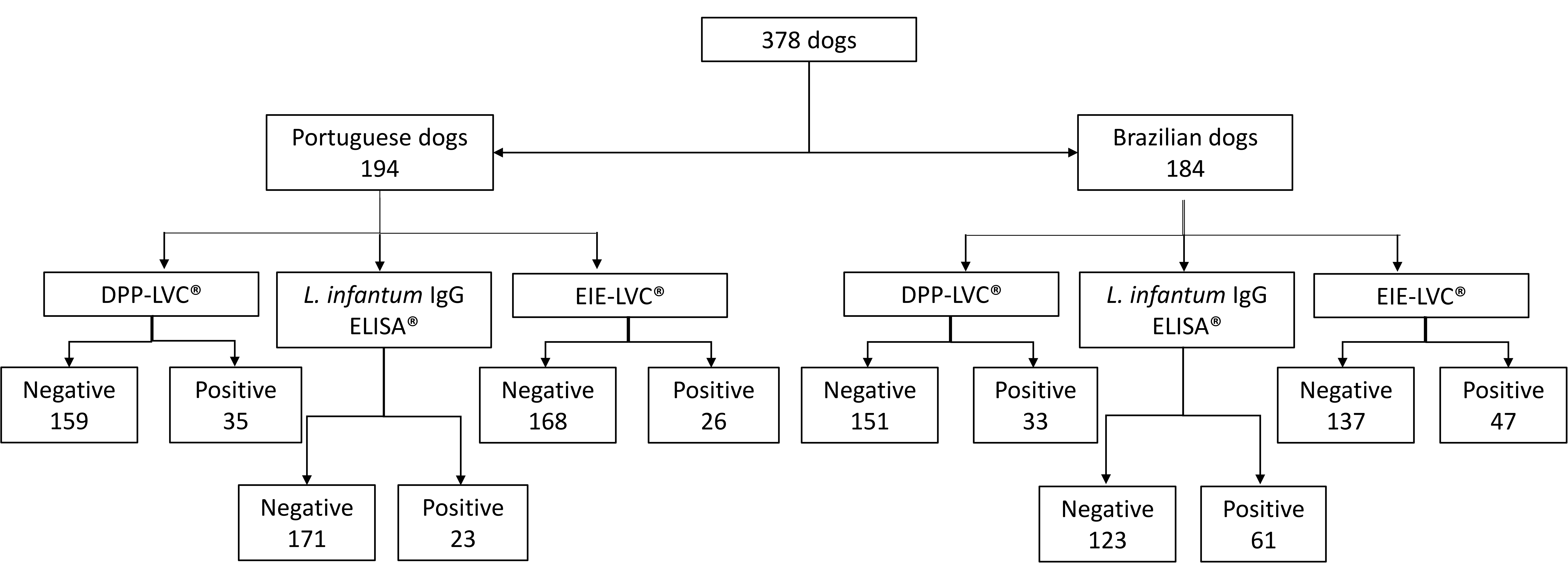

Supplement: Supplementary file 4 — Additional file 4: Figure S1. Flowchart of the participants according to standards for reporting the diagnostic accuracy of the serodiagnostic techniques tested in the present study using Bayesian latent class models. [file 13071_2022_5328_MOESM4_ESM.tif]
